# The Utility of Rapid On-Site Evaluation during Bronchoscopic Biopsy: A 2-Year Respiratory Endoscopy Central Experience

**DOI:** 10.1155/2019/5049248

**Published:** 2019-12-08

**Authors:** Hansheng Wang, Na Wei, Yijun Tang, Yunyun Wang, Guoshi Luo, Tao Ren, Chang Xiong, Hongbo Li, Meifang Wang, Xin Qian

**Affiliations:** ^1^Department of Pulmonary and Critical Care Medicine, Taihe Hospital, Hubei University of Medicine, Shiyan, 442000 Hubei, China; ^2^Respiratory Endoscopy Center, Taihe Hospital, Hubei University of Medicine, Shiyan, 442000 Hubei, China; ^3^Department of Cardiothoracic Surgery, Taihe Hospital, Hubei University of Medicine, Shiyan, 442000 Hubei, China

## Abstract

**Background:**

Rapid on-site evaluation (ROSE) is commonly used to evaluate the adequacy of biopsy materials in fine-needle aspiration; however, the diagnostic performance of ROSE during fiber optic bronchoscopy (FOB) biopsy under direct vision is rarely reported. Here, we evaluated the role of ROSE during FOB biopsy of visible lesion in trachea or bronchi.

**Methods:**

The role of ROSE was prospectively evaluated in consecutive bronchoscopy specimens obtained between January 2016 and January 2018. The agreement and accuracy between ROSE and final histopathological interpretation were assessed. The frequency and possible reasons for discrepancy between ROSE and definitive histopathology results were identified. Histological and cytological classification was performed according to the International Association for the Study of Lung Cancer, the American Thoracic Society, and the European Respiratory Society (IASLC/ATS/ERS) criteria of lung ADCs classification.

**Results:**

The study enrolled 651 patients, of which 33 were excluded because of insufficient cells. Final diagnosis of malignancy was achieved in 462 cases (74.8%), whereas 156 cases (25.2%) were nonmalignant. ROSE and pathology were well correlated for the diagnosis of squamous cell carcinoma (SCC) (Kappa = 0.718, *p* < 0.05), adenocarcinoma (AdC) (Kappa = 0.662; *p* < 0.05) and small cell lung cancer (SCLC) (Kappa = 0.955; *p* < 0.05). In 24 cases diagnosed as malignant by ROSE and nonmalignant by pathology, the lesion tissues were surgically excised and re-analyzed, and the 24 cases were finally confirmed as malignant by pathology.

**Conclusions:**

ROSE technique allows bronchoscopists to obtain viable and adequate material for the diagnosis of histopathology, and provides them with an onsite preliminary diagnosis especially in cases with inconclusive macroscopic appearance. ROSE and pathology should be used in combination to increase the accuracy of diagnosis.

## 1. Background

In recent years, rapid on-site evaluation (ROSE) has been rapidly popularized in endobronchial ultrasound-guided transbronchial needle aspiration (EBUS-TBNA) [1–5], or fine needle aspiration biopsy (FNA) [6–8] for assessing materials' adequacy and categorizing diagnosis during biopsy procedure. Chandra et al. [9] and Nakajima et al. [2] demonstrated that ROSE is critical for assessing the adequacy of cytological smears and cytology is comparable to histology in the diagnosis of lung lesions. In addition, Fassina et al. [8] reported a satisfactory overall agreement of 71.4% was achieved in differentiating the cancer histological types in fine needle aspiration (FNA) for ROSE, and Nakajima et al. [2] reported a concordance rate of ROSE and final pathologic diagnosis of 94.3% in endobronchial ultrasound-guided transbronchial needle aspiration (EBUS-TBNA).

The use of ROSE may allow an earlier termination of the procedure based on the confirmation that diagnostic tissue has been retrieved, and may, therefore, improve diagnostic sensitivity by redirecting sampling to adjacent sites in the event of a negative ROSE finding [10, 11]. In addition, ROSE can help bronchoscopists determine whether additional specimen needs to be collected for further ancillary studies, such as immunochemistry, molecular detection, and microbiology [8]. Visible lesions are biopsied under direct vision using fiber optic bronchoscopy (FOB); however, the quality and adequacy of biopsy materials are not known. Because ROSE has good intermodality agreement with histopathology and can provide the bronchoscopist with an onsite preliminary diagnosis, it may solve these problems. However, the utility of ROSE during bronchoscopy biopsy under direct vision is rarely reported, and so its role has remained unclear. We conducted this prospective randomized study to further clarify the role of ROSE in assessing materials' adequacy and categorizing diagnosis during biopsy procedure.

## 2. Materials and Methods

All 651 patients who underwent bronchial biopsy combined with ROSE in TaiHe Hospital (Shiyan, Hubei, China) were enrolled in the study. Informed consent was obtained from all patients, and FOB biopsy was performed by a bronchoscopist. Study was approved by the Taihe Hospital Ethics Committees. ROSE was performed in the presence of a cytopathologist in the procedural room of bronchoscopy when possible, cytopathologist immediately interpreted whether the sample was negative (no malignant cells) or positive (definitive cytopathologic evidence of malignancy or special infection evidence). A small portion of each biopsy pass was placed on a slide, smeared, and stained with Diff-Quik for onsite analysis. Slide preparations were performed by the procedural pulmonologist or by an assistant who was a member of the procedural team. A smear was considered adequate if it contained an abundancy of preserved cells or showed sufficient cellularity compatible with the clinical and/or radiological findings. Inadequate material was subjected to re-biopsy instantaneously. The tissue was fixed with formalin and sent to the pathology department for immunohistochemical stains and molecular studies. We categorized the histological and cytological subtypes in accordance with the IASLC/ATS/ERS [12]. Our diagnoses include definitely adenocarcinoma (AC), favor AC, definitely SqCC, favor SqCC, and NSCLC-NOS, we classified definitely adenocarcinoma (AC) and favor AC into AC, and definitely SqCC, favor SqCC into SqCC in our research. The diagnosis was made on the basis of histologic characteristics—clear-cut evidence of pearl formation, keratinization for SqCC differentiation, and glandular structure for AC differentiation, then IHC evaluation staining intensity was graded as focal and weak, diffuse and strong, negative. Final diagnosis was determined with p63, TTF-1, and neuroendocrine immunostain for all cases. p63(+) was accepted for an SCC, TTF-1(+) was accepted for an ADC. Diagnosis was assigned as adenosquamous cell carcinoma, when both p63 and TTF-1 are positive. If cytology was NSCLC-NOS but histology was SqCC or AC, the criterion for final diagnosis was accepted on the basis of histology. If histology was NSCLC-NOS but cytology was SqCC or AC, the final diagnosis was accepted on the basis of histology also. If IHC was not available, histology and final diagnosis was NSCLC-NOS.

When granulomatous inflammation with necrosis was observed during ROSE, biopsy specimen was considered sufficient and supported for tuberculosis diagnosis by ROSE. After that histopathology, Zeihl–Neelson (ZN) stains combined with PCR were performed for final diagnosis of tuberculosis. Trachea and bronchi fungal infections are mainly aspergillus in our study, visible aspergillus hyphae provide evidence for cytology or histopathology diagnosis, microbial cultures of specimen was needed.

### 2.1. Statistical Analysis

Receiver operator characteristic (ROC) curves were designed to assess sensitivity and specificity for the estimated parameters. Chi-square test was used to compare diagnostic accuracy rates between the disease specific groups. Intermodality agreement between ROSE results and final histopathological diagnosis was assessed by calculating a κ-score. Probability values <5% (*p* < 0.05) were considered statistically significant.

## 3. Results

### 3.1. Cases

Between January 2017 and January 2019, 651 cases were enrolled, of which 502 were men and 149 were women. The median age was 58.7 years (range, 13–84 years). During bronchoscopic examination, of 651 patients, 284 (43.6%) showed visible bronchial neoplasms and 367 (56.4%) showed bronchial mucosal lesions, as shown in Table 1.

### 3.2. Onsite Evaluation

**Figure 1:**
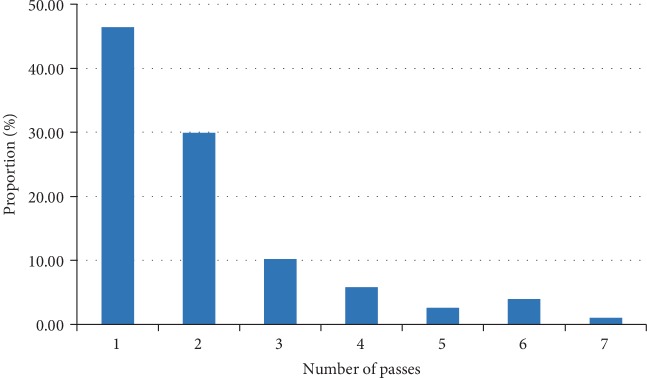
Biopsy pass adequate materials obtained during ROSE.

Of the 651 smears, 618 (94.9%) were adequate and 33 (5.1%) were inadequate because of excessive blood or necrosis. Among the 618 satisfactory cases, 46.4% (287/618) were adequate on the first biopsy pass, 30% (185/618) on the second pass, and 10.2% (63/618) on the third pass. The remaining cases included 5.8% (36/618), 2.5% (16/618), 4% (25/618), and 1% (6/618) obtained during the fourth, fifth, sixth, and seventh passes, respectively. An average of two biopsy passes was required to obtain adequate specimens during ROSE (Figure 1).

### 3.3. Diagnostic Categories of Malignancy

Good overall consistency was achieved in categorizing the histological types of malignancy (344/438; 78.5%) (Table 2). There were 126 cases of squamous cell carcinoma (SCC) confirmed by ROSE and pathology; ROSE and pathological diagnosis results were consistent (Kappa = 0.718, *p* < 0.05), with a PPV of 74.1%, NPV of 92.8%, specificity of 86.0%, and sensitivity of 85.7%. For adenocarcinoma (AC), ROSE and pathological diagnosis were consistent (Kappa = 0.662, *p* < 0.05), with a PPV of 69.6%, NPV of 93.7%, specificity of 89.3%, and sensitivity of 80.6%. In small cell lung cancer (SCLC), ROSE and pathological diagnosis were well correlated (Kappa = 0.955, *p* < 0.05), with a PPV of 95.9%, NPV of 99.2%, specificity of 98.0%, and sensitivity of 95.3%. In addition, for the same biopsy specimen, there were 35 cases of ROSE and pathology consistently diagnosed as NSCLC-NOS. However, ROSE showed poor diagnostic accuracy for specific tumors such as adenoid cystic carcinoma (ACC), mucoepidermoid carcinoma (MEC), sarcomatoid carcinoma (SARC), and fetal lung adenocarcinoma (FLAC). For metastases, ROSE could only identify suspicious findings, whereas it could not distinguish the source of metastasis. As shown in Table 1, all nonmalignant cases diagnosed by histopathology were considered as malignancy by ROSE, which identified 16 cases of SCC, seven cases of AC, and one small cell carcinoma case. These 24 cases were finally diagnosed as malignant tumors by computed tomography (CT)-guided percutaneous lung biopsy, pleural biopsy, TBNA, or surgical specimen biopsy, which confirmed 16 cases of SCC, seven cases of AC, and one case of small cell carcinoma.

### 3.4. Benign Diagnosis

Tuberculosis was diagnosed in 57 patients, of which 46 showed a consistent diagnosis by ROSE (Kappa = 0.814, *P* < 0.001). Diagnostic specificity and sensitivity were 80.7% and 98.0%, respectively. The area under the ROC curve of ROSE for the diagnosis of tuberculosis was 0.893 (95% CI: 0.83–0.96, *P* < 0.001). Of five cases of mycosis confirmed by pathology, three were correlated with ROSE (Kappa = 0.744, *P* < 0.001), the area under the ROC curve of ROSE for the diagnosis of mycosis was 0.80 (95% CI: 0.00–1.00, *P* < 0.001). For acute/chronic nonspecific inflammation, Cohen's kappa demonstrated a satisfactory agreement of 0.815 (*P* < 0.001) between histopathology and cytology as shown in Table 3.

Table 4 shows the cytopathological correlation and diagnostic accuracy in various lung lesions. The diagnostic accuracy of ROSE was 89.4% for SCC, 80.6% for AC, and 95.3% for SCLC, similar to that of histology.

## 4. Discussion

In this study, we demonstrate that ROSE ensures the quality of biopsy specimens during FOB procedure, and the results of ROSE correlated well with the final pathological diagnosis based on hematoxylin and eosin (H&E) staining of biopsy samples of FOB.

Although technical advances in histopathology have reduced the processing time for small biopsies to a few hours [13], cytology remains the method of choice for an immediate assessment of sample adequacy, whereas biopsies can be used for different purposes [14]. The proportion of inadequate specimens reportedly ranges from 2.7% to 14.3% during FNA biopsy [6, 15, 16], consistent with our finding of 5.1% (33/651) inadequacy. Koul et al. [17] reported that only 11% of cases show adequate materials on first FNA, and an average of 3.5 biopsy passes required to obtain adequate material during FNA; however, our data suggested that 46.6% of the cases had adequate materials on first biopsy, 76.6% had adequate materials on second biopsy, and 86.6% satisfactory samples were obtained on third biopsy. A possible reason for this result was that lesions could not be visualized directly during FNA, and ultrasound or imaging was needed for detecting the location and size of lesions. Conversely, lesions were visible during FOB biopsy, and an experienced bronchoscopist detected suspicious lesions directly.

Considering patients' safety and cost effectiveness, studies suggest that ROSE can quickly evaluate the adequacy of TBNA or FNA samples [15, 18, 19], eliminate unnecessary biopsies, help reduce costs, and avoid or reduce complications [11, 20, 21]. The present study showed that using FOB combined with ROSE, adequate specimens were obtained in 86.6% of patients in 3 biopsies, suggesting that this regimen is appropriate for FOB biopsy.

**Figure 2:**
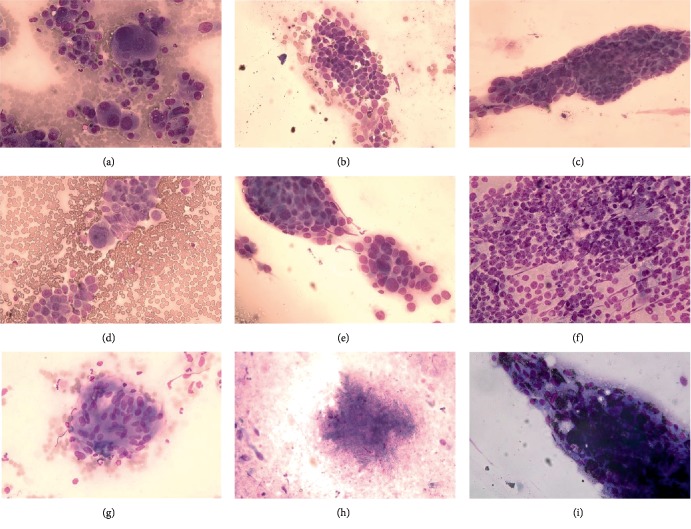
*Cytological characteristics of ROSE.*(a) Well-and moderately differentiated adenocarcinoma of acinar type with obvious glandular differentiation (Diff-quick). (b) Poorly differentiated adenocarcinoma shows dyscohesive aggregate cells with large nuclei, prominent nucleoli, and tumor cells with single intracytoplasmic vacuoles or globular secretory material indicate glandular differentiation (Diff-quick). (c) Well-differentiated squamous cell carcinoma: smears composed of mainly dispersed, often elongated or spindle-shaped cells with dense cytoplasm and keratinization, nuclei are often pyknotic or hyperchromatic with angulated contours (Diff-quick). (d) Moderately differentiated squamous cell carcinoma showing small dyscohesive sheet of malignant cells with enlarged nuclei with nucleoli and coarse chromatin and fragments or dispersed keratinizing cells with dense cytoplasm and pyknotic nuclei. (e) Poorly differentiated squamous cell carcinoma showing large tissue fragments composed of cells with enlarged nuclei with macronucleoli (Diff-quick). (f) Small cells with high N/C ratio, crush artifacts scant and poorly preserved cytoplasm apoptosis, “salt and pepper” chromatin texture, and nuclear molding fit well with small cell carcinoma (Diff-quick). (g) Granulomatous with necrosis, or visible langhans cells are typical features of tuberculosis in ROSE (Diff-quick). (h) The diameter of mycosis is generally 7–10 *μ*m, the division of mucor is right angle bifurcation or hyphae are not separated, aspergillus appears as acute angle bifurcation, with necrotic background and neutrophils (Diff-quick). (i) After the alveolar phagocytic cells phagocytose the dust, they evolve into dust cells, which show that the cytoplasm contains abundant black carbon particles.

Whether lung lesions can be accurately diagnosed using ROSE remains controversial [9]. Therefore, in the present study, we combined bronchial FOB biopsy with ROSE, and analyzed the diagnostic accuracy of specimens for benign and malignant lung diseases. Chandra et al. [9] compared diagnosis by bronchial lavage fluid, brush, TBNA, and percutaneous needle aspiration biopsy specimens by ROSE, and showed that ROSE cytology and histology were comparable, and ROSE may be superior to histopathology for the diagnosis of lung tumors. In the present study, evidence of malignancy was not obtained by histology in 24 lung cancer patients, whereas ROSE detected cancer cells in these patients. Further analysis by CT-guided percutaneous lung puncture, thoracoscopic pleural biopsy, and TBNA confirmed the presence of lung cancer in the 24 patients. One possible reason for this discrepancy is that biopsy specimens contain fewer malignant tissue components, whereas they are sufficient for cytological smears. Alternatively, pathological sections may have failed to effectively obtain lesion tissue. Ravaioli et al. [22] showed that TBNA combined with ROSE is useful for the rapid diagnosis of lung cancer, as it not only allows evaluation of the adequacy of TBNA needle biopsy specimens, but also enables the classification of lung cancer [23, 24]. Celik et al. reported that ROSE has high diagnostic yield over subclassification of NSCLC-NOS [24], and cytology, with or without cell block, successfully subclassifies NSCLC-NOS cases and can be a substitute for IHC in resource-poor laboratories and in low-income countries [23]. In our present study, there was good agreement between ROSE and final pathology for SCLC. The diagnostic accuracy of 96.8% was in agreement with reports by Fassina et al. [8] and Ravaioli et al. [22], who reported an overall accuracy for small cell carcinoma of 90–97%. In addition, the ROSE results for SCC and AC were consistent with the final pathological diagnosis, but with a low Kappa value. Poorly differentiated SCC or AC, however, displays some features that may be confused with other poorly differentiated neoplasms [25]. It is not always possible based on morphology alone to distinguish these neoplasms from poorly differentiated neoplasms in limited samples [26]. The characteristics of small cell carcinoma cells are more obvious, as cancer cells are arranged in a row and crowded, the cytoplasm is smaller or even absent, and the nucleus has a salt and pepper appearance, with a nuclear model (Figure 2); therefore, the diagnostic accuracy and pathological consistency are better.

Reactive changes in bronchiolar epithelium might be sometimes pronounced, making it difficult to distinguish these from malignant epithelial cells, but reactive atypia in the bronchiole epithelium is characterized by loose sheets of bronchiolar cells with moderate nuclear enlargement, irregularity, and hyperchromasia, Usually, these atypical reactive cells occur as a few dispersed cells or few small cell clusters, which helps to avoid a diagnosis of malignancy during ROSE [27, 28]. In addition, there is a difference between alveolar phagocytes and lung adenocarcinoma, pulmonary macrophages with abundant foamy cytoplasm and frequent intracytoplasmic carbon particles [27, 28]. Adenocarcinoma with signet ring, adenocarcinoma is the most common primary lung tumor and displays a variety of morphological features. Most tumors have acinar, papillary, or micropapillary features, although the acinar type is the most common [27, 28].

The features of tuberculosis under the microscope include granulomatous inflammation, necrosis, mixed lymphocytes and epithelial-like cells, and Langhans giant cells [29], sarcoidosis characterized by nonnecrotizing epithelioid cell granulomas and different from granulomatous inflammation of tuberculosis [28]. For Aspergillus and Cryptococcus, accurate diagnosis is possible if the fungus is identified. Therefore, fungal and tuberculosis infections can be accurately diagnosed onsite by experienced pathological cytologists, thus gaining time for timely clinical treatment. Although ROSE has many advantages, there are also disadvantages such as the need for a professionally trained cytopathologist on site [9].

In conclusion, ROSE can provide guidance for the bronchoscopist to obtain adequate lesion specimens during FOB biopsy and can provide an onsite preliminary diagnosis especially in cases showing an inconclusive macroscopic appearance. Because ROSE and pathology have their own advantages and disadvantages, they can complement each other to improve the accuracy of diagnosis.

## 5. Conclusions

In this study, we demonstrated that ROSE technique allows bronchoscopists to obtain viable and adequate material for the diagnosis of histopathology, and provides them with an onsite preliminary diagnosis especially in cases with inconclusive macroscopic appearance. ROSE and pathology should be used in combination to increase the accuracy of diagnosis.

## Figures and Tables

**Table 1:** Characteristics of patients and lesions.

Number of patients	651
Gender (male/female)	502/149
Age (years) (mean ± SD)	58.7 ± 11.2
*Type of lesion*(%)	
Neoplasm	43.6% (284/651)
Mucosal lesion	56.4% (367/651)

**Table 2:** Correlation of ROSE with pathology in malignant cases (*n* = 462).

ROSE	Histopathology								
	SCC	AC	NSCLC	SCLC	Suspicious cancer	Other malignancy	Metastasis	Non malignant	Total
SCC	126	9	6	3	4		6	16	170
AC	10	87	6	0	3	1 (FLAC)	11	7	125
NSCLC	6	3	35	0	4	1 (SARC)			49
									
SCLC	0	3	0	94			0	1	98
Suspicious cancer	5	6	2			2 (MEC/ACC)	3	0	18
Other malignancy						2 (Lymphoma)			2
Metastasis	0	0	0	0	0		0	0	0
Non malignant	0	0	0	0	0		0	0	0
Total	147	108	49	97	11	6	20	24	462

**Table 3:** Correlation of ROSE with pathology in benign cases (*n* = 156).

ROSE	Final diagnosis					
	Tuberculosis	Mycosis	Granulomatous	Acute/chronic nonspecific inflammation	Atypical hyperplasia	Total, no. (%)
Tuberculosis	46		1	1		48 (30.8)
Mycosis		3				3 (1.9)
Granulomatous	1		1			2 (1.3)
Acute/chronic nonspecific inflammation	9	2	2	86		99(57.0)
Atypical hyperplasia	1				3	4 (2.6)
Total, no. (%)	57 (36.5)	5 (3.2)	4 (2.6)	87 (55.8)	3 (1.9)	156

**Table 4:** Cyto-histopathological correlation in lung lesions.

Diagnosis	ROSE(number of cases)	Histology (number of cases)	Final diagnosis (number of cases)	Diagnostic accuracy of histology (%)	Diagnostic accuracy of ROSE (%)
SCC	167	147	163	90.2	87.1
AC	125	108	115	93.9	81.7
NSCLC	52	49	52	100	71.4
SCLC	98	97	95	98.9	96.9
Other malignancy	2	6	6	100	33.3
Metastasis	0	20	20	100	0
Tuberculosis	48	57	57	100	84.2
Mycosis	3	5	5	100	60
Granulomatous	2	4	4	100	50
Acute/chronic nonspecific inflammation	86	87	87	100	98.9
Atypical hyperplasia	3	3	3	100	100

## Data Availability

The datasets used and/or analysed during the current study are available from the corresponding author on reasonable request.

## Abbreviations

ROSE: Rapid on-site evaluation
FOB: Fiber optic bronchoscopy
SCC: Squamous cell carcinoma
AdC: Adenocarcinoma
SCLC: Small cell lung cancer
EBUS-TBNA: Endobronchial ultrasound-guided transbronchial needle aspiration
FNA: Fine needle aspiration biopsy
ROC: Receiver operator characteristic
ACC: Adenoid cystic carcinoma
MEC: Mucoepidermoid carcinoma
SARC: Sarcomatoid carcinoma
FLAC: Fetal lung adenocarcinoma
CT: Computed tomography.
